# β-glucan from *Lentinus edodes* inhibits breast cancer progression via the Nur77/HIF-1α axis

**DOI:** 10.1042/BSR20201006

**Published:** 2020-12-14

**Authors:** Xiuru Zhang, Tingting Li, Shuwen Liu, Yiming Xu, Minjun Meng, Xiumin Li, Zhichao Lin, Qici Wu, Yu Xue, Yutian Pan, Gulimiran Alitongbieke

**Affiliations:** 1 Engineering Technological Center of Mushroom Industry, Minnan Normal University, Zhangzhou, Fujian 363000, People’s Republic of China; 2 Vascular Department Zhongshan Hospital of Xiamen University, Xiamen, Fujian 363000, People’s Republic of China

**Keywords:** β-glucan of Lentinus edodes, Breast cancer, Hypoxia, Hypoxia-induced factor-1α, Nuclear receptor Nur77

## Abstract

**Background:** β-glucan from *Lentinus edodes* (LNT) is a plant-derived medicinal fungus possessing significant bioactivities on anti-tumor. Both hypoxia-induced factor-1α (HIF)-1α and Nur77 have been shown to be involved in the development of breast cancer. However, there is yet no proof of Nur77/HIF-1α involvement in the process of LNT-mediated tumor-inhibition effect.

**Methods:** Immunohistochemistry, immunofluorescence and Hematoxylin–Eosin staining were used to investigate tumor growth and metastasis in MMTV-PyMT transgenic mice. Proliferation and metastasis-associated molecules were determined by Western blotting and reverse transcription-quantitative PCR. Hypoxic cellular model was established under the exposure of CoCl_2_. Small interference RNA was transfected using Lipofectamine reagent. The ubiquitin proteasome pathway was blunted by adding the proteasome inhibitor MG132.

**Results:** LNT inhibited the growth of breast tumors and the development of lung metastases from breast cancer, accompanied by a decreased expression of HIF-1α in the tumor tissues. In *in vitro* experiments, hypoxia induced the expression of HIF-1α and Nur77 in breast cancer cells, while LNT addition down-regulated HIF-1α expression in an oxygen-free environment, and this process was in a manner of Nur77 dependent. Mechanistically, LNT evoked the down-regulation of HIF-1α involved the Nur77-mediated ubiquitin proteasome pathway. A strong positive correlation between Nur77 and HIF-1α expression in human breast cancer specimens was also confirmed.

**Conclusion:** Therefore, LNT appears to inhibit the progression of breast cancer partly through the Nur77/HIF-1α signaling axis. The findings of the present study may provide a theoretical basis for targeting HIFs in the treatment of breast cancer.

## Introduction

Breast cancer is one of the most frequently diagnosed cancers worldwide with a high mortality rate [1]. According to statistics from 2012, 1.67 million women worldwide were diagnosed with breast cancer, accounting for 25.2% of all cancers in women [2]. As the increasing breast cancer incidence in women is alarming, there is an urgent need for more effective methods of prevention, diagnosis and treatment. Traditional cancer therapies, such as surgery, chemotherapy, and radiation therapy, exhibit various limitations due to poor prognosis and severe associated side effects [3]. Currently, natural product extracts appear to hold promise as a therapeutic strategy due to their ability to prevent and treat cancer [4]. Polysaccharides isolated from natural sources, such as fungi, plants, algae and animals, have been extensively investigated and found to possess anti-tumor activity over the past few decades [5,6]. Therefore, anti-cancer drugs discovered and developed from natural resources are expected to become a focus anti-cancer drug intervention study.

Orphan nuclear receptor Nur77, a product of an immediate early gene, is also referred to as TR3, nuclear receptor subfamily 4 group A member 1 (NR4A1) and nerve growth factor-induced clone B (NGFI-B) [7]. Nur77 has the typical structural characteristics of nuclear receptors, including the activation functions (AF)-1, DNA-binding domain (DBD), ligand-binding domain (LBD), and AF-2 domains, but no specific *in vivo* ligands have been identified. However, it can respond to a number of intracellular and extracellular signaling stimuli, for example, it may participate in the body’s energy regulation, and can regulate cell proliferation, cell differentiation, cell apoptosis, mitosis, autophagy and senescence, among others [8]. Related research indicates that Nur77 plays a dual role for the development of tumors [9]. Nur77 promotes invasion and metastasis of breast cancer and colorectal cancer (CRC) by activating transforming growth factor-β, matrix metallopeptidase (MMP)-9 and E-cadherin [10,11]. On the other hand, overexpression of Nur77 also inhibits cancer cell and tumor growth of other types of solid tumors [12,13]. There is increasing evidence that Nur77 is a key regulator in immune responses, such as T-cell dysfunction, nuclear factor-κB-dependent inflammatory signaling and macrophage polarization [14,15]. In the hypoxic microenvironment, macrophages accumulation and polarization contribute to tumor angiogenesis and creation of an inflammatory environment which is associated with hypoxia-inducible factor (HIF)-1α (HIF-1α). Importantly, multiple interactions between HIF-1α and Nur77 have been found during numerous tumors growth. It is confirmed that overexpression of the potent oncogenic protein β-catenin in CRC can increase Nur77 transcription through HIF-1α rather than T-cell factor, suggesting that there is an interaction between Nur77 and HIF-1α during tumorigenesis [16].

β-glucan from *Lentinus edodes* (LNT) (a β-1,3-glucan) is a type of lentinan containing two β-1,6-glucose branches, and is the first medicinal fungus to enter the field of modern biotechnology. LNT appears to exert marked antitumor effects due to its unique triple-helical conformation [17]. Clinical studies have demonstrated that chemical immunotherapy with lentinan may prolong the survival of patients with advanced gastric cancer compared with chemotherapy alone [18]. Since the 1970s, extensive research has shown that lentinan alone or in combination with other chemotherapeutic drugs may be used to treat ovarian [19], gastric [18], liver [20] and lung [21] cancers. As an immunomodulator, the underlying anti-tumor mechanism may be the activation of immune responses to induce cell apoptosis [22]. Of note, α- and β-d-glucans from *Shiitake mushrooms* have been recently demonstrated to be effective on MDA-MB-231 breast carcinoma cells, whereas no cytotoxic activity on MCF-10A cells, considered as a normal mammary gland cell counterpart [23]. Furthermore, the impact of LNT mycelial extract on breast cancer therapy has recently been reviewed including breast cancer cell lines, tumor bearing animals and clinical trials [24]. A few studies are only dedicated to reveal the molecular mechanisms underlying LNT anti-cancer effect. However, it is not known whether LNT-mediated tumor suppression in breast cancer involves the interaction between Nur77 and HIF-1α.

The aim of the present study was to investigate whether LNT administration suppresses the growth and metastasis of breast cancer by inhibiting HIF-1α, and whether this effect is Nur77-dependent. The results may provide a novel insight into the mechanism underlying LNT-induced tumor suppression, and indicate whether β-glucan or foods containing β-glucan may be used for treating the patients with lung metastases from breast cancer.

## Materials and methods

### Subjects

A total of six breast cancer patients (three patients with invasive ductal breast carcinoma, grade II; two with invasive ductal breast carcinoma, grade III; one patient with papillary carcinoma) from Zhongshan Hospital of Xiamen University were included in the present study (ranging from March to June 2019). Clinical information is shown in Table 1. Informed consent was signed and the experimental protocol was approved by the Human Ethics Committee of Zhongshan Hospital of Xiamen University (ethic number: 2018027). Briefly, clinical tumor samples (0.4 cm^3^) and paired corresponding adjacent normal tissue were collected and cut into pieces from the mentioned six patients. Adjacent normal tissue was obtained on the set of 2 cm from tumor edge. Then the tissues were lysed using RIPA buffer through tissue homogenate. After standing for 30 min on ice, the cell lysate was centrifuged at 12000 rpm for 10 min. The supernatant was collected and 40 μg protein were used to performed Western blotting.

**Table 1:** Clinical information of breast cancer patients

Number	Age	Gender	IHC	TNM stage	Pathological diagnosis
477111	68	Female	ER (+ +), PR (+ +), Her-2 (1+), TOPI-II (+/-), E-Cad (++), CK-L (++), CD56 (-), CgA (-), SYN (-), Ki-67 (+, 3%)	T3 N1 M1	Invasive ductal breast carcinoma (II)
460482	40	Female	ER (+ +), PR (+ +), Her-2 (2+), TOPI-II (+, 8%), E-Cad (+), P120 (+), Ki-67 (+, 7%)	T3 N1 M1	Invasive ductal breast carcinoma (III)
470667	44	Female	ER (+ +), PR (+ +), Her-2 (2+), CK5/6 (-), SMA (+), calponin (+), p63 (+), CD10 (+), Ki-67 (+, 20%)	T1 N0 M0	Papillary carcinoma
465297	49	Female	ER (+ +), PR (+ +), Her-2 (0), TOPI-II (+, 3%), E-Cad (++), P120 (++), Ki-67 (+, 10%)	T2 N1 M0	Invasive ductal breast carcinoma (II)
465298	41	Female	ER (+ +), PR (+ +), Her-2 (1+), TOPI-II (+, 2%), E-Cad (++), P120 (++), Ki-67 (+, 4%)	T2 N1 M0	Invasive ductal breast carcinoma (II)
470354	31	Female	ER (+, 50%), PR (+, 30%), Her-2 (3+), TOPI-II (+, 10%), Ki-67 (+, 60%)	T2 N0 M0	Invasive ductal breast carcinoma (III)

### Animals and treatment

MMTV-PyMT transgenic mice (FVB), which are well-recognized breast cancer model mice, were purchased from The Jackson Lab. All animals were housed at 20 ± 2°C, with a 12-h dark–light cycle and a relative humidity of 60–70%. The mice had *ad libitum* access to normal chow and water under specific pathogen-free (SPF) conditions. All animal experiments were approved by the Animal Ethics Committee of Minnan Normal University and Xiamen University (ethic number: IACUC-20190311-01). After genotyping through PCR amplification using specific primer of PyMT gene (Supplementary Figure S1), 8-week-old female MMTV-PyMT transgenic mice with similar initial body weight and growth status were selected and randomly divided into two groups, the control and drug-treated groups (*n*=6/group). Mice were killed in the following situations, for example: during the treatment with LNT, mice lost 15% weight or more of their body weight or mice continued to suffer pain from tumor burden such as tumor diameter reaching to more than 2.5 centimeters and ulcerated tumor. Tumor growth of all ten mammary glands were evaluated by biweekly palpations and measured by Vernier calliper from days 21 to 70. Tumor onset was recorded as the first day when tumors could be detected by palpation. Tumor size was calculated and categorized into two groups randomly. The control group was injected with a solvent that dissolves the drug. LNT was isolated from the dried fruiting bodies of *Lentinus edodes* (LNT) according to previously reported methods. In brief, the fruiting bodies were immersed in boiling water (100°C) for 2 h followed by the immersion of 1.25 M NaOH/0.05% NaBH_4_ at room temperature for two-times. After centrifugation to remove the residues, the supernatant was collected and performed hyperfiltration. Then the entrapped sugar was filtered by sepharoseA50 and washed with 0.02 M Tris (pH: 7.2) and Tris solution containing 0.3% NaOH. The filter liquor was then hyperfiltrated and centrifugated to remove the residues and subsequently with freeze-drying. The drying product was Lentinan (LNT). The product and purity were identified by High Performance Liquid Chromatography-mass spectrometer analysis (HPLC/MS) according to previous study [6] (Supplementary Figure S2). Briefly, combined with phenol–sulfuric acid analysis using d-glucose and the method of Bradford using bovine serum albumin (BSA, Sigma), total sugar composition of LNT and protein content was analyzed to prove that LNT used in the following experiments was of high purity. The endotoxin contained in LNT was assessed by the chromogenic limulus amebocyte lysate assay according to previous study [25]. LNT was identified as a β-(1,3)-glucan with β-(1,6)-branches by GC-MS and NMR. The mean molecular weight of LNT was conducted to be 562.8474 kDa by HPLC. Finally, LNT was dissolved in PBS buffer for *in vitro* assays and in saline solution for *in vivo* assays.

For the treatment of mice, LNT was administered by tail intravenous injection at a dose of 20 mg/kg for 2 consecutive weeks in the drug-treated group. At the end of the administration, all mice were killed using cervical dislocation method and the tumor tissues and lung tissues were immediately harvested. Each mouse presented ten subcutaneous tumors (one tumor per mammary gland). Tumor tissue samples were photographed and weighed. And the long diameter of each mice at the time of killing was recorded. Subsequently, part of the tumor tissue and lung tissue were used for protein detection and some were stored at −80°C for frozen sections. In addition, a portion of the sample was immersed in 4% paraformaldehyde and embedded in paraffin. The selected tissues in the follow-up experiments were guaranteed to be the same site of the mouse mammary solid tumor tissue. The parallelism of the materials was guaranteed, and the remaining samples were stored in liquid nitrogen.

### Cell culture, transfection and cell treatment

The human breast cancer cell lines MCF-7 and T47D were obtained from the Type Culture Collection of the Chinese Academy of Sciences (Shanghai, China). The cells were cultured in high-glucose Dulbecco’s modified Eagle’s medium (DMEM) supplemented with 1% penicillin/streptomycin and 10% (v/v) fetal bovine serum (FBS) and maintained at 37°C in a 5% CO_2_ incubator. MCF-7 cells were seeded in 60-mm culture dishes. After 24 h of pre-incubation, cells were transfected with siRNA and scramble siRNA of Nur77 (sc-36109, Santa Cruz Biotechnology, Inc.) using Lipofectamine 3000 (Invitrogen; Thermo Fisher Scientific, Inc.) according to the manufacturer’s protocol. The sequence of Nur77 siRNA was as follows: 5′-GGC UUG AGC UGC AGA AUG A-3′. At 48 h post-transfection, the cells were rinsed with PBS and stimulated with LNT. For oxygen-deficient environment, cells were treated with 100 μM CoCl_2_ (Sigma–Aldrich, St. Louis, U.S.A.) for different times (0.5, 1, 3, 6 9, 12, 24, 36 and 48 h). We also used 20 μM MG132 (Selleck, U.S.A.) to block the ubiquitin proteasome pathway (treatment for 6 h) under low oxygen conditions. Cells were seeded in 6- or 12-well plates at a density of 10^5^ cells/cm^2^ the day prior to the experiments. Cell lysates were collected for subsequent Western blotting analysis.

### Histological staining

Mouse lung tissue samples were placed in 4% paraformaldehyde at 4°C, then dehydrated with graded ethanol, immersed in xylene, and embedded in paraffin. The paraffin was cut into 4-μm longitudinal sections. Following dewaxing, the sections were stained with Hematoxylin–Eosin (HE) according to the manufacturer’s instructions (Beyotime Institute of Biotechnology, China). Each group of samples was observed with an optical microscope (Olympus IX71, Olympus Corporation). For immunohistochemical staining, endogenous peroxidase activity was blocked with 0.3% hydrogen peroxide at room temperature for 30 min. Nonspecific protein binding was blocked by incubation with 2% BSA for 20 min and then incubated with the primary antibody anti-vascular endothelial growth factor (VEGF) polyclonal antibody (dilution 1:100; Affinity) or anti-HIF-1α (diluted 1:100; Affinity) overnight at 4°C. Subsequently, the sections were incubated with secondary antibody for another 1 h at room temperature (Beijing Zhongshan Biotechnology, China). The sections were counterstained for 1 min by using Hematoxylin. Each group were examined using a light microscope (Olympus IX71; Olympus Corporation).

### Immunofluorescence staining

Paraffin sections (4 μm) of tumor and lung tissue were subjected to immunofluorescence staining. Primary antibody against proliferating cell nuclear antigen (PCNA; dilution 1:100; Affinity) was used to detected the proliferation ability. The Alexa Fluor 488-labeled goat anti-rabbit IgG (dilution 1:200; Molecular Probes) was used as the secondary antibody. DAPI (dilution 1:300; Beyotime Institute of Biotechnology) was used to stain the nucleus. Photograph observation was performed under a Biological inverted microscope (IX51, Olympus, Japan). Comprehensive analysis included measuring staining intensity and the number of positive cells using ImagePro Plus software (Media Cybernetics, Inc., Maryland, U.S.A.). Five high-power fields for each sample were chosen for evaluation by three independent pathologists.

### Western blotting

Total protein of breast cancer cells and tumor tissue were extracted using lysis buffer. Briefly, the cells were seeded at a density of 3 × 10^6^ cells/dish in a 60-mm culture dish in a medium containing 10% FBS for 24 h and then treated with or without LNT under different conditions. At the end of the treatment, cells were harvested and treated with RIPA buffer containing 1 mM PMSF for 20 min on ice. Following centrifugation at 12000 rpm for 15 min, the supernatant was saved as a total protein extract. Tumor tissue was cut into small pieces and lysed on ice using the same RIPA buffer as above for 20 min. After centrifugation at 12000 rpm for 15 min, the supernatant was collected as a tumor tissue protein extract. The membrane was incubated with corresponding specific primary antibodies against VEGF, HIF-1α, β-actin, MMP9, N-cadherin, α-smooth muscle actin (SMA), Nur77, PCNA, poly (ADP) ribose polymerase (PARP), p-Akt, AKT, p-mammalian target of rapamycin (mTOR) and mTOR. After incubating with the appropriate horseradish peroxidase-conjugated secondary antibodies (dilution, 1:2000; Beyotime Institute of Biotechnology) for 1 h at room temperature, blots were detected with Super Signal West Femto Maximum Sensitivity Substrate (Thermo Fisher Scientific, Inc.) and visualized by Versa Doc (Bio-Rad Laboratories, Inc.). The optical densities of the protein bands were quantified by Quantity One software (Bio-Rad, Laboratories, Inc.) and normalized to the optical density of GAPDH or β-actin on the same membrane.

### Statistical analysis

All experiments were processed at least three times. All data were expressed as mean ± standard deviation. Quantitative analysis of intracellular proteins was proceeded by Quantity One software and statistical analysis was determined by two-tailed Student’s *t* test using GraphPad Prism software (GraphPad Software, Inc.). *P*<0.05 was considered to indicate statistically significant differences.

## Results

### LNT inhibits tumor biological characteristics along with the decrease in HIF-1α in MMTV-PyMT transgenic mice

**Figure 1:**
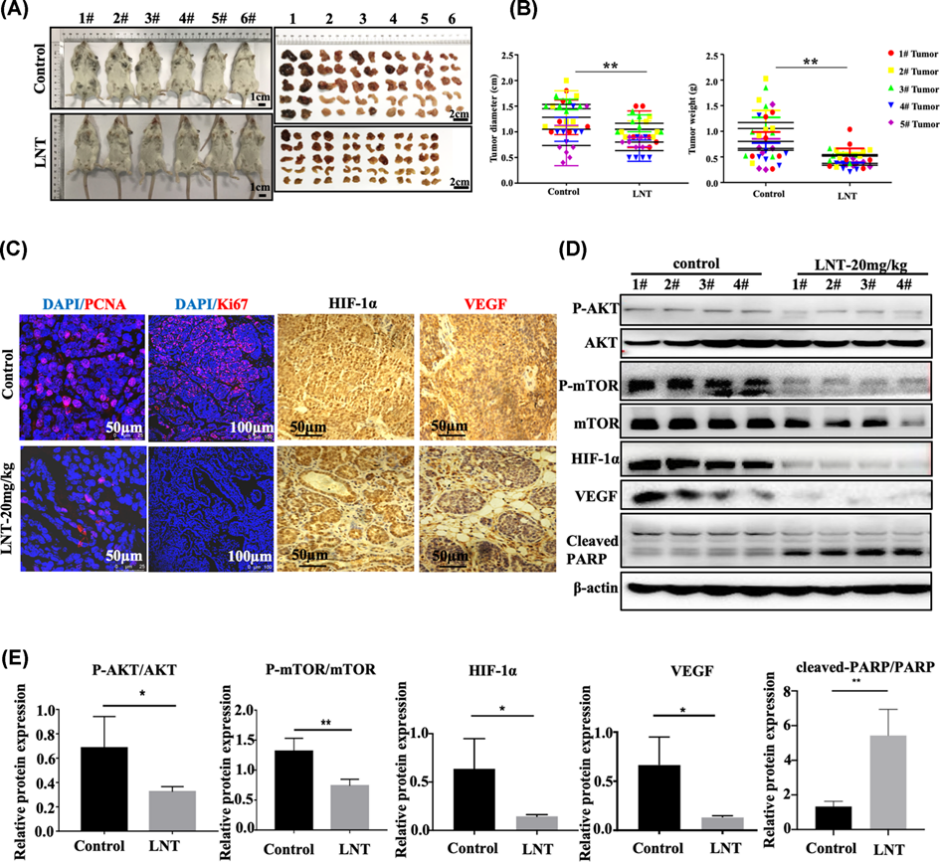
Effects of LNT on breast tumor growth and expression of HIF-1α in mice (**A**) Mammary tumor size observation in the LNT group and the control group. (**B**) Tumor weight statistics for the LNT and control groups. (**C**) Immunofluorescence was used to determine the expression of PCNA and Ki67 in LNT group and control group. Immunohistochemistry was employed to detect the expression of HIF-1α and VEGF in both groups. (**D**) The expression of VEGF, HIF-1α, PARP, phosphorylated Akt, Akt, phosphorylated mTOR and mTOR in different groups as analyzed by Western blotting. β-Actin served as the internal control. (**E**) Quantitative analysis of each protein in panel (D). Abbreviation: LNT, *Lentinus edodes*. Data are shown as the means ± standard deviation (SD), *n*≥3. *P<0.05; **P<0.01.

LNT was reported to inhibit the proliferation of breast cancer cells [26], but whether HIF-1α was involved in this process remained unknown. To solve this problem, MMTV-PyMT transgenic mice that displayed obvious breast tumor contour characteristics at 2 months of age were employed for *in vivo* experiments. In the present study, LNT treatment significantly suppressed the growth of breast tumors, followed by the declined body weight in MMTV-PyMT transgenic mice compared with that in the control group (Figure 1A and Supplementary Figure S3 and Table S1). Meanwhile, the maximum diameter of each mice and the tumor weight in each pair of mammary glands in the LNT-treated group were notably lower compared with that in the control mice (Figure 1B and Supplementary Tables S2 and S3). Microscopically, the decrease in PCNA and Ki67 expression were observed in LNT-exposed tumor tissues (Figure 1C, the first row). These results indicate that LNT obviously suppressed the tumor growth of breast cancer.

Importantly, the number of HIF-1α- and VEGF-positive cells in tumor tissues was significantly reduced following LNT addition (IHC staining results) (Figure 1C, the second and third rows). Through Western blotting, it also confirmed that LNT not only reduced the levels of VEGF and HIF-1α in tumor tissues, but also inhibited the activity of Akt and mTOR (Figure 1D,E). Further investigation revealed that LNT administration promoted cleavage of the apoptotic protein PARP (Figure 1D,E). These data indicate that LNT inhibits tumor tissue growth, proliferation and its downstream signal transduction, accompanied by a reduction in HIF-1α expression. The inhibitory effect of LNT on tumor growth may be mediated by apoptosis activation.

### LNT inhibits lung metastasis of breast tumors in MMTV-PyMT mice

**Figure 2:**
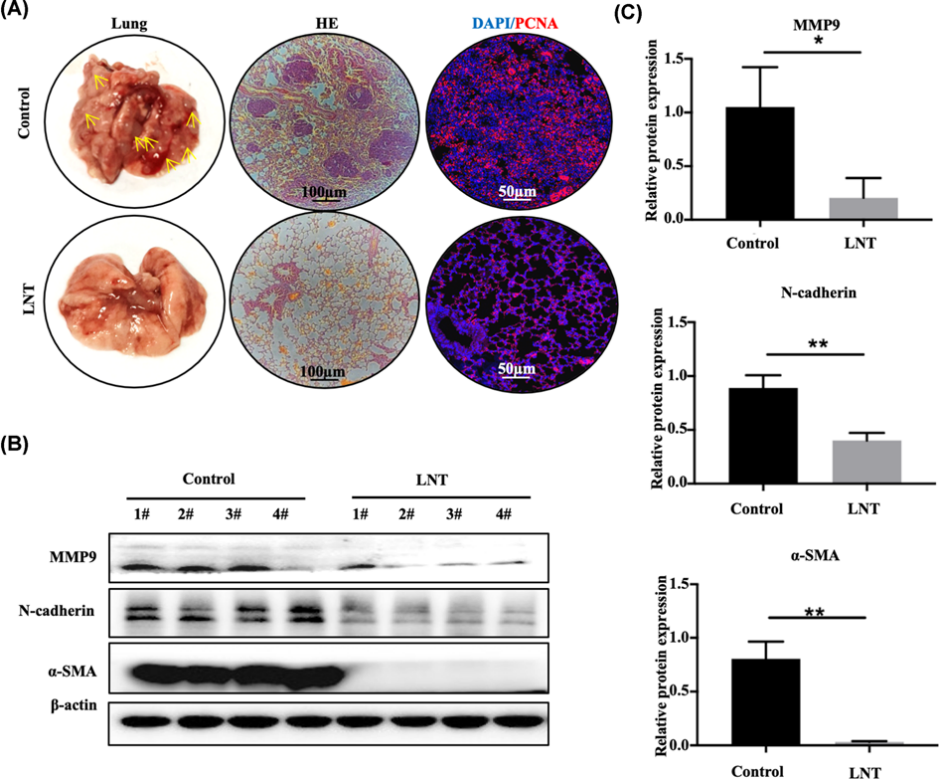
Impact of LNT on lung metastasis of mouse breast tumors (**A**) Tumor images (left) and HE staining results of lung tissue in the LNT group and control group (middle). The expression of PCNA was detected by immunofluorescence in the LNT group and control groups (right). Yellow arrows: tumor mass. (**B**) The expression of MMP9, N-cadherin and α-SMA proteins in different groups as analyzed by Western blotting. (**C**) Quantitative analysis of each protein in panel (B). Abbreviation: LNT, *Lentinus edodes*. Data are shown as the means ± SD, *n*≥3. *P<0.05; **P<0.01.

MMTV-PyMT transgenic mice develop lung metastasis from breast cancer. To explore the role of LNT in lung metastasis, tumor images and HE staining demonstrated that multiple inflammatory lesions appeared in the lung tissues of the control group (Figure 2A, the left row). However, only few inflammatory lesions were observed in the lung tissue of mice treated with LNT (Figure 2A). Subsequent immunofluorescence staining in lung tissue indicated that the number of PCNA-positive cells in LNT-exposed mice was significantly lower compared with that of the control group (Figure 2A). Additionally, the expressions of migration-related proteins MMP9, N-cadherin, and α-SMA were significantly higher in the control group compared with the LNT group (Figure 2B,C). The above results demonstrate that LNT effectively counteracts the metastasis ability of breast cancer cells to the lung.

### LNT down-regulates the expression of HIF-1α and Nur77 under the hypoxic condition

**Figure 3:**
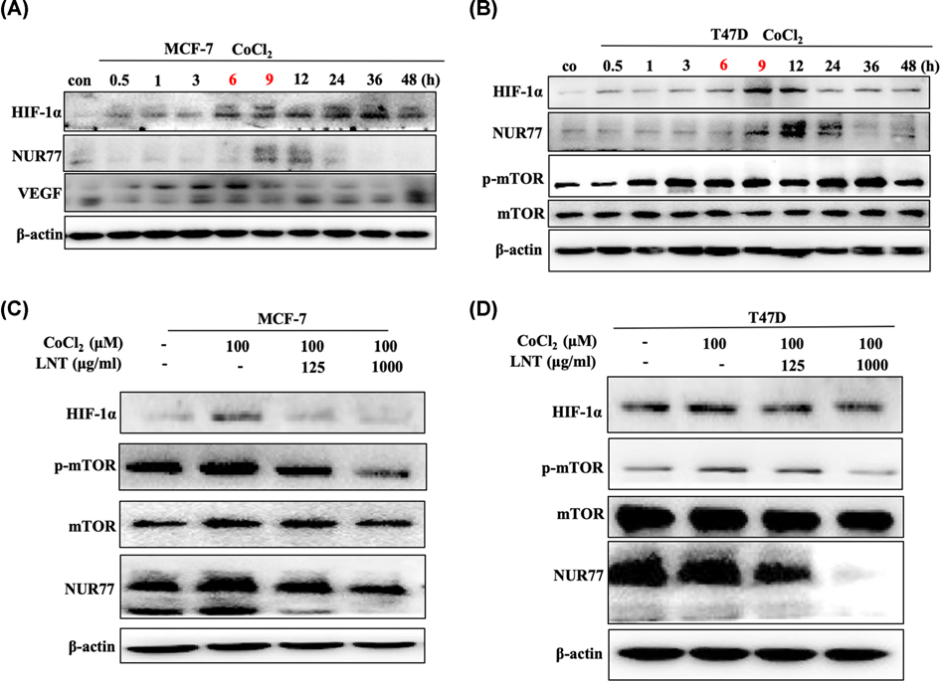
Association of HIF-1α and Nur77 in breast cancer cells under anoxic conditions Protein expression of HIF-1α, Nur77 and VEGF (**A**) in MCF-7 cells and HIF-1α, Nur77 and p-mTOR (**B**) in T47D cells treated with cobalt chloride for 0, 0.5, 1, 3, 6, 9, 12, 24, 36 and 48 h. Protein expression of HIF-1α, Nur77 and mTOR (**C**) in MCF-7 cells and (**D**) T47D cells treated with different concentrations of LNT under treatment with cobalt chloride (0 and 100 μM). Abbreviation: LNT, *Lentinus edodes*. Data are shown as the means ± SD, *n*≥3.

Next, MCF-7 cells and T47D cells were employed to further elucidate whether HIF-1α participated in the process of LNT-mediated breast cancer suppression. Related reports indicate that with the prolongation of hypoxia stimulation, the expression of HIF-1α initially increases, and then declines [27]. In our research, it was observed that the expression of HIF-1α and VEGF increased with the treatment time of cobalt chloride, and reached a maximum at 9 h in MCF-7 cells (Figure 3A and Supplementary Figure S4A). After continued culture, the expression levels of HIF-1α and VEGF were gradually reduced (Figure 3A and Supplementary Figure S3A). Interestingly, after cobalt chloride treatment, Nur77 expression increased transiently at 9 and 12 h, and also exhibited a decreasing trend with prolonged treatment time (Figure 3A and Supplementary Figure S3A). T47D cells also displayed an initially increasing trend in HIF-1α and Nur77 expression, followed by a decrease under conditions of oxygen deficit. Additionally, hypoxia also induced the up-regulation of mTOR activity in T47D cells (Figure 3B and Supplementary Figure S4B), indicating that hypoxia would continuously affect the inflammatory signaling in tumor cells. Once treated with LNT, hypoxia induced the up-regulation of HIF-1α/Nur77 and mTOR activity was obviously neutralized in MCF-7 and T47D cells, and the neutralization was concentration-dependent (Figure 3C,D and Supplementary Figure S4C,D).

### LNT-mediated HIF-1α inhibition is involved in the Nur77-dependent ubiquitin proteasome pathway

**Figure 4:**
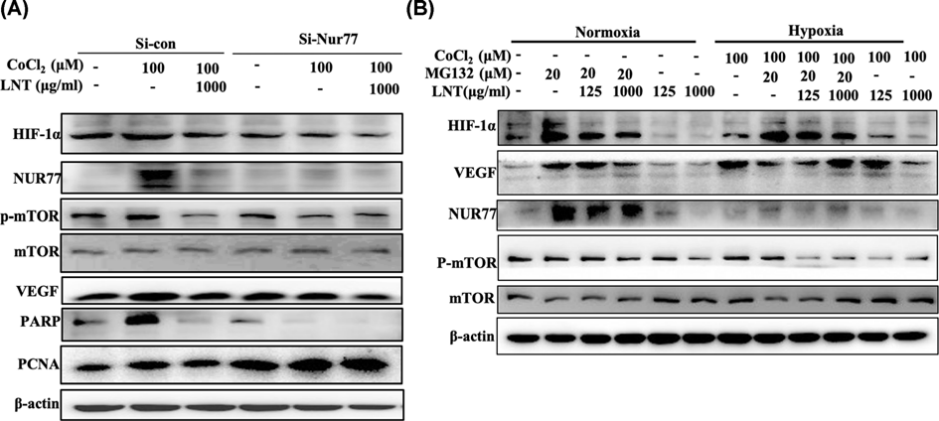
Role of ubiquitin proteasome pathway in LNT-mediated degradation of HIF-1α (**A**) Expression of HIF-1α, Nur77, mTOR, VEGF, PARP and PCNA in MCF-7 cells treated with cobalt chloride (0 and 100 μM) in the control group and Nur77-knockdown group. (**B**) Protein levels of HIF-1α, Nur77, mTOR and VEGF as analyzed by Western blotting in MCF-7 cells treated with MG132 and LNT under normoxic and hypoxic conditions. Abbreviation: LNT, *Lentinus edodes*r. Data are shown as the means ± SD, *n*≥3.

To further investigate whether the effect of LNT on HIF-1α depended on Nur77, small interference RNA of Nur77 was used to knock down its expression in breast cancer cells (Supplementary Figure S5). It was found that down-regulation of Nur77 not only inhibited the expression of HIF-1α, but also reduced the expression of VEGF and PCNA, mTOR activity and full-length PARP under oxygen-deficit conditions (Figure 4A and Supplementary Figure S6). Conversely, LNT addition repressed hypoxia-activated mTOR/Nur77/HIF-1α and its downstream signaling molecules including PCNA and PARP in cells transfected with negative control. Once Nur77 was depleted, the neutralizing effect of LNT on this signaling pathway was abrogated, indicating that LNT launched the degradation of HIF-1α was Nur77-dependent in breast cancer cells (Figure 4A and Supplementary Figure S6).

The degradation of HIF-1α is involved in the ubiquitin proteasome pathway [28]. To explore the mechanism through which LNT degraded HIF-1α, proteasome inhibitor MG132 was employed to treat breast cancer cells under stimulation by cobalt chloride and LNT. Under normoxia, MG132 addition activated (Figure 4B, second row vs first row; and Supplementary Figure S7), while LNT inhibited the HIF-1α/Nur77 signaling axis and VEGF expression (Figure 4B, fifth and sixth rows vs first row; and Supplementary Figure S7) in MCF7 cells. However, this inhibition induced by LNT was abolished after MG132 treatment (Figure 4B, third and fourth rows VS second row; and Supplementary Figure S7). Under hypoxic conditions, HIF-1α, Nur77 and VEGF expressions were significantly increased compared with that cells under normoxic conditions (Figure 4B, seventh row vs first row; and Supplementary Figure S7). However, MG132 further promoted HIF-1α and Nur77 expression, but partially inhibited VEGF expression under hypoxia conditions (Figure 4B, eighth row vs seventh row; and Supplementary Figure S7). As previously mentioned, LNT negatively regulated HIF-1α/Nur77/VEGF signaling axis in hypoxia environment (Figure 4B, eleventh and twelfth rows vs seventh row; and Supplementary Figure S7). Of note, in the absence of oxygen, MG132 abrogated the LNT-mediated the degradation of HIF-1α and the decline of VEGF expression, but did not affect Nur77 level (Figure 4B and Supplementary Figure S7). It was also observed that neither MG132 nor LNT, or their combination treatment, had no effect on mTOR activity in normoxia. Conversely, LNT significantly inhibited mTOR activity in hypoxia environment, which was not reversed in the presence of MG132 (Figure 4B and Supplementary Figure S7). The above results demonstrate that LNT launched the degradation of HIF-1α via Nur77 may depend on the ubiquitin proteasome pathway, and this effect is independent of oxygen content.

### There is a positive correlation between Nur77 and HIF-1α expression in breast cancer patients

**Figure 5:**
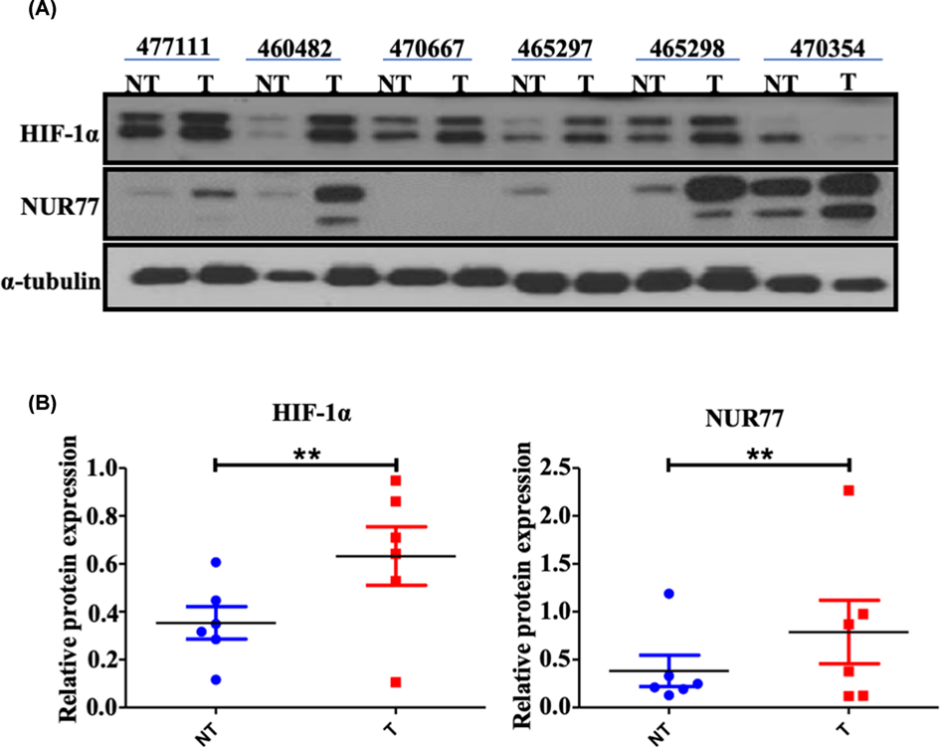
Correlation between Nur77 and HIF-1α in breast cancer patients (**A**) Protein expression of HIF-1α and Nur77 in six clinical breast cancer patients as determined by Western blotting. (**B**) Scatter plot of quantitative analysis of each protein in panel (A). Abbrevition: HIF, hypoxia-inducible factor. Data are shown as the means ± SD, *n*≥3.

To further investigate the association of HIF-1α and Nur77, six clinical breast tumor tissues were collected from three patients with invasive ductal breast carcinoma (grade II), two patients with invasive ductal breast carcinoma (grade III) and one patient with papillary carcinoma. The clinical information of the patients was summarized in Table 1. It was found that the expression of HIF-1α in the tumor tissues of the five groups (83.33%) was significantly higher than that in the adjacent tissues, and the expression of Nur77 in the four tumor tissues (66.67%) was significantly higher compared with that in the adjacent tissues (Figure 5A,B). Among the tissues, high expression of both HIF-1α and Nur77 appeared in the tumor tissues of three patients (numbers 1, 2 and 5) compared with adjacent tissues (Figure 5A). Thus, there appears to be a positive correlation between Nur77 and HIF-1α expression in breast tumor tissues, which also supports that the mechanism underlying the progression of breast cancer may involve the Nur77/HIF-1α signaling axis.

## Discussion

Tumor hypoxia is a common finding in breast cancer and is associated with a significant increase in the risk of tumor cell metastasis and patient mortality [29]. Studies have shown that the hypoxic microenvironment contributes to the development of breast cancer [29]. Hypoxia induces breast cancer stem cell phenotype by HIF-dependent [30]. Therefore, targeted hypoxia intervention is crucial for breast tumor growth. LNT was proven to regulate HIF-1α in order to inhibit the growth of breast tumors, which provides an important reference for the treatment of breast cancer.

Currently, accumulating evidence indicates that tumor hypoxia is a principal character in breast cancer which is closely associated with a significant increase in the risk of tumor cell metastasis, growth, invasion and patient mortality [31]. Hypoxia stimulates tumor angiogenesis and metastasis through up-regulating HIF-1α, followed by VEGF activation [32]. HIF-1α is overexpressed in primary breast cancer, that is associated with over proliferation and poor differentiation [33,34]. However, whether the regulation of breast cancer progression by LNT has the involvement of HIF-1α remains elusive. In the present study, LNT treatment inhibited the growth of breast tumor along with a declined VEGF and HIF-1α levels, and the decreased AKT and mTOR activities. Related studies have shown that β-glucan can interfere with the process of tumor lung metastasis from colon cancer or B16-BL6 melanoma cells in a dose-dependent manner [35]. Our results also revealed that there were few inflammatory lesions in the lung tissue of the LNT group, implying that LNT inhibited the capability of breast tumor cells to invade to lung tissue. Hypoxic microenvironment is the hallmark of solid tumors. HIF-1α expression is able to modulate the recruitment of macrophages to tumor hypoxic areas, leading to strong immune responses [36]. Accumulating evidence shows that LNT is a critical switch on immunoregulation in tumor development; thus, it may be hypothesized that LNT-induced decreased HIF-1α is partly through tumor immunosuppression.

HIF-1 is a transcription factor that widely exists in mammals and humans under anoxic conditions [37]. Under normal conditions, the α subtype has a half-life of no more than 5 min and is then degraded. During hypoxia, stabilization of HIF-1α occurs by inhibiting 4-prolyl hydroxylase activity, an enzyme that requires oxygen to function [38]. It has been reported that HIF-1α specifically binds to the promoter of Nur77 and subsequently activates the transcription of Nur77 under hypoxic stimulation [39]. Our previous findings have also confirmed that Nur77 can stabilize the expression of HIF-1α in hypoxic state of ovarian cancer cells (unpublished data). Here, in breast cancer cells, the expression of HIF-1α and Nur77 also increased instantaneously under hypoxic conditions. Of note, LNT addition inhibited hypoxia-mediated the accumulation of HIF-1α in a concentration-dependent and Nur77-dependent manner. In addition to that, the strong association between Nur77 and HIF-1α was also observed in breast cancer specimens. Recently, Nur77 has been proven to be a key immunomodulator and its inhibition may be a promising strategy for cancer immunotherapy [40]. As mentioned above, HIF-1α also participates in the process of tumor immune responses. Given that LNT-mediated HIF-1α inhibition depended on Nur77, it may be inferred that the anti-tumor effect of LNT on breast cancer is through regulating immune response via the Nur77/HIF-1α axis.

Nur77 up-regulation is able to inhibit the binding of mouse double minute 2 (MDM2) to HIF-1α, leading to the degradation of HIF-1α by the ubiquitin proteasome pathway [41]. Nur77 also stabilizes HIF-1α by inhibiting pVHL-mediated HIF-1α ubiquitination [42]. This study found that ubiquitin-proteasome inhibitor blocked LNT-evoked the degradation of HIF-1α under hypoxia conditions, but did not significantly affect LNT-mediated the inhibition in Nur77 expression. Therefore, LNT-mediated down-regulation of HIF-1α under treatment is possibly dependent on the ubiquitin proteasome pathway. However, what role of Nur77 in this ubiquitin proteasome pathway needs a further exploration.

Taken together, the results of the present study demonstrate that LNT inhibits the growth of breast tumors in mice and the ability to metastasize to lung tissues, which is accompanied by the decline of HIF-1α in tumor tissues. *In vitro*, LNT down-regulates hypoxia-induced HIF-1α in a concentration-dependent and Nur77-dependent manner, which is possibly mediated by the ubiquitin proteasome pathway. Our results explain the LNT-mediated mechanism underlying breast tumor suppression, and may provide a theoretical basis for targeting HIFs in the treatment of breast cancer.

## Supplementary Material

Supplementary Figures S1-S7 and Tables S1-S3

## Data Availability

The data used to support the findings of the present study are included within the article.

## Abbreviations

AF: activation function
BSA: bovine serum albumin
CRC: colorectal cancer
DAPI: 4′,6-diamidino-2-phenylindole
GAPDH: Glyceraldehyde 3-phosphate dehydrogenase
FBS: fetal bovine serum
HE: Hematoxylin and Eosin
HIF-1α: hypoxia-induced factor-1α
LNT: β-glucan from *Lentinus edodes*
MMP: matrix metallopeptidase
MMTV-PyMT: Mouse Mammary Tumor Virus-Polyoma Middle T Antigen
mTOR: mammalian target of rapamycin
PARP: poly (ADP) ribose polymerase
PCNA: proliferating cell nuclear antigen
SMA: α-smooth muscle actin
VEGF: vascular endothelial growth factor

## References

[1] Ferlay J., Steliarova-Foucher E., Lortet-Tieulent J., Rosso S., Coebergh J.W., Comber H. et al. (2013) Cancer incidence and mortality patterns in Europe: estimates for 40 countries in 2012. Eur. J. Cancer 49, 1374–1403 10.1016/j.ejca.2012.12.027 23485231

[2] Ferlay J., Soerjomataram I., Dikshit R., Eser S., Mathers C., Rebelo M. et al. (2015) Cancer incidence and mortality worldwide: sources, methods and major patterns in GLOBOCAN 2012. Int. J. Cancer 136, E359–E386 10.1002/ijc.29210 25220842

[3] Siegel R., Naishadham D. and Jemal A. (2012) Cancer statistics for Hispanics/Latinos, 2012. CA Cancer J. Clin. 62, 283–298 10.3322/caac.21153 22987332

[4] Nagalingam A., Kuppusamy P., Singh S.V., Sharma D. and Saxena N.K. (2014) Mechanistic elucidation of the antitumor properties of withaferin a in breast cancer. Cancer Res. 74, 2617–2629 10.1158/0008-5472.CAN-13-2081 24732433 PMC4009451

[5] Vannucci L., Krizan J., Sima P., Stakheev D., Caja F., Rajsiglova L. et al. (2013) Immunostimulatory properties and antitumor activities of glucans (Review). Int. J. Oncol. 43, 357–364 10.3892/ijo.2013.1974 23739801 PMC3775562

[6] Xu H., Zou S., Xu X. and Zhang L. (2016) Anti-tumor effect of beta-glucan from Lentinus edodes and the underlying mechanism. Sci. Rep. 6, 28802 10.1038/srep28802 27353254 PMC4926123

[7] Pekarsky Y., Hallas C., Palamarchuk A., Koval A., Bullrich F., Hirata Y. et al. (2001) Akt phosphorylates and regulates the orphan nuclear receptor Nur77. Proc. Natl. Acad. Sci. U.S.A. 98, 3690–3694 10.1073/pnas.051003198 11274386 PMC31113

[8] Beard J.A., Tenga A. and Chen T. (2015) The interplay of NR4A receptors and the oncogene-tumor suppressor networks in cancer. Cell. Signal. 27, 257–266 10.1016/j.cellsig.2014.11.009 25446259 PMC4276441

[9] Niu G., Lu L., Gan J., Zhang D., Liu J. and Huang G. (2014) Dual roles of orphan nuclear receptor TR3/Nur77/NGFI-B in mediating cell survival and apoptosis. Int. Rev. Cell Mol. Biol. 313, 219–258 10.1016/B978-0-12-800177-6.00007-4 25376494

[10] Zhou F., Drabsch Y., Dekker T.J., de Vinuesa A.G., Li Y., Hawinkels L.J. et al. (2014) Nuclear receptor NR4A1 promotes breast cancer invasion and metastasis by activating TGF-beta signalling. Nat. Commun. 5, 3388 10.1038/ncomms4388 24584437

[11] Wang J.R., Gan W.J., Li X.M., Zhao Y.Y., Li Y., Lu X.X. et al. (2014) Orphan nuclear receptor Nur77 promotes colorectal cancer invasion and metastasis by regulating MMP-9 and E-cadherin. Carcinogenesis 35, 2474–2484 10.1093/carcin/bgu157 25064356

[12] Lee S.O., Abdelrahim M., Yoon K., Chintharlapalli S., Papineni S., Kim K. et al. (2010) Inactivation of the orphan nuclear receptor TR3/Nur77 inhibits pancreatic cancer cell and tumor growth. Cancer Res. 70, 6824–6836 10.1158/0008-5472.CAN-10-1992 20660371 PMC2988472

[13] Wu J.P., Liu J., Jia R.P. and Song H.B. (2013) Nur77 inhibits androgen-induced bladder cancer growth. Cancer Invest. 31, 654–660 10.3109/07357907.2013.853077 24299210

[14] Popichak K.A., Hammond S.L., Moreno J.A., Afzali M.F., Backos D.S., Slayden R.D. et al. (2018) Compensatory expression of Nur77 and Nurr1 regulates NF-kappa B-dependent inflammatory signaling in astrocytes. Mol. Pharmacol. 94, 1174–1186 10.1124/mol.118.112631 30111648 PMC6117504

[15] Liu X.D., Wang Y., Lu H.P., Li J., Yan X.W., Xiao M.L. et al. (2019) Genome-wide analysis identifies NR4A1 as a key mediator of T cell dysfunction. Nature 567, 525–+ 10.1038/s41586-019-0979-8 30814730 PMC6507425

[16] To S.K., Zeng W.J., Zeng J.Z. and Wong A.S. (2014) Hypoxia triggers a Nur77-beta-catenin feed-forward loop to promote the invasive growth of colon cancer cells. Br. J. Cancer 110, 935–945 10.1038/bjc.2013.816 24423919 PMC3929893

[17] Li S., Huang Y., Wang S., Xu X. and Zhang L. (2014) Determination of the triple helical chain conformation of beta-glucan by facile and reliable triple-detector size exclusion chromatography. J. Phys. Chem. B 118, 668–675 10.1021/jp4087199 24400948

[18] Ina K., Kataoka T. and Ando T. (2013) The use of lentinan for treating gastric cancer. Anticancer Agents Med. Chem. 13, 681–688 10.2174/1871520611313050002 23092289 PMC3664515

[19] Fujimoto K., Tomonaga M. and Goto S. (2006) A case of recurrent ovarian cancer successfully treated with adoptive immunotherapy and lentinan. Anticancer Res. 26, 4015–4018 17195451

[20] Zhang Y., Li Q., Wang J., Cheng F., Huang X., Cheng Y. et al. (2016) Polysaccharide from Lentinus edodes combined with oxaliplatin possesses the synergy and attenuation effect in hepatocellular carcinoma. Cancer Lett. 377, 117–125 10.1016/j.canlet.2016.04.037 27130669

[21] Liu W., Gu J., Qi J., Zeng X.N., Ji J., Chen Z.Z. et al. (2015) Lentinan exerts synergistic apoptotic effects with paclitaxel in A549 cells via activating ROS-TXNIP-NLRP3 inflammasome. J. Cell. Mol. Med. 19, 1949–1955 10.1111/jcmm.12570 25858687 PMC4549045

[22] Xu H., Zou S.W., Xu X.J. and Zhang L.N. (2016) Anti-tumor effect of beta-glucan from Lentinus edodes and the underlying mechanism. Sci. Rep. 6, 1–13 10.1038/srep28802 27353254 PMC4926123

[23] Morales D., Rutckeviski R., Villalva M., Abreu H., Soler-Rivas C., Santoyo S. et al. (2020) Isolation and comparison of alpha- and beta-D-glucans from shiitake mushrooms (Lentinula edodes) with different biological activities. Carbohydr. Polym. 229, 1–12 10.1016/j.carbpol.2019.115521 31826486

[24] Wong J.H., Ng T.B., Chan H.H.L., Liu Q., Man G.C.W., Zhang C.Z. et al. (2020) Mushroom extracts and compounds with suppressive action on breast cancer: evidence from studies using cultured cancer cells, tumor-bearing animals, and clinical trials. Appl. Microbiol. Biotechnol. 104, 4675–4703 10.1007/s00253-020-10476-4 32274562

[25] Lindsay G.K., Roslansky P.F. and Novitsky T.J. (1989) Single-step, chromogenic limulus amebocyte lysate assay for endotoxin. J. Clin. Microbiol. 27, 947–951 10.1128/JCM.27.5.947-951.1989 2745704 PMC267460

[26] Xu H., Zou S.W. and Xu X.J. (2017) The beta-glucan from Lentinus edodes suppresses cell proliferation and promotes apoptosis in estrogen receptor positive breast cancers. Oncotarget 8, 86693–86709 10.18632/oncotarget.21411 29156828 PMC5689718

[27] Almholt K., Juncker-Jensen A., Laerum O.D., Dano K., Johnsen M., Lund L.R. et al. (2008) Metastasis is strongly reduced by the matrix metalloproteinase inhibitor Galardin in the MMTV-PymT transgenic breast cancer model. Mol. Cancer Ther. 7, 2758–2767 10.1158/1535-7163.MCT-08-0251 18790756

[28] Oh E.T., Kim J.W., Kim J.M., Kim S.J., Lee J.S., Hong S.S. et al. (2016) NQO1 inhibits proteasome-mediated degradation of HIF-1 alpha. Nat. Commun. 7, 1–14 10.1038/ncomms13593 PMC5171868 27966538

[29] Semenza G.L. (2016) The hypoxic tumor microenvironment: a driving force for breast cancer progression. Biochim. Biophys. Acta 1863, 382–391 10.1016/j.bbamcr.2015.05.036 26079100 PMC4678039

[30] Zhang C., Samanta D., Lu H., Bullen J.W., Zhang H., Chen I. et al. (2016) Hypoxia induces the breast cancer stem cell phenotype by HIF-dependent and ALKBH5-mediated m(6)A-demethylation of NANOG mRNA. Proc. Natl. Acad. Sci. U.S.A. 113, E2047–E2056 10.1073/pnas.1602883113 27001847 PMC4833258

[31] Ryan H.E., Lo J. and Johnson R.S. (1998) HIF-1 alpha is required for solid tumor formation and embryonic vascularization. EMBO J. 17, 3005–3015 10.1093/emboj/17.11.3005 9606183 PMC1170640

[32] Jensen R.L., Ragel B.T., Whang K. and Gillespie D. (2006) Inhibition of hypoxia inducible factor-1alpha (HIF-1alpha) decreases vascular endothelial growth factor (VEGF) secretion and tumor growth in malignant gliomas. J. Neuro Oncol. 78, 233–247 10.1007/s11060-005-9103-z 16612574

[33] Zhong H., De Marzo A.M., Laughner E., Lim M., Hilton D.A., Zagzag D. et al. (1999) Overexpression of hypoxia-inducible factor 1alpha in common human cancers and their metastases. Cancer Res. 59, 5830–5835 10582706

[34] Bos R., Zhong H., Hanrahan C.F., Mommers E.C.M., Semenza G.L., Pinedo H.M. et al. (2001) Levels of hypoxia-inducible factor-1 alpha during breast carcinogenesis. J. Natl. Cancer Inst. 93, 309–314 10.1093/jnci/93.4.309 11181778

[35] Yoon T.J., Kim T.J., Lee H., Shin K.S., Yun Y.P., Moon W.K. et al. (2008) Anti-tumor metastatic activity of beta-glucan purified from mutated Saccharomyces cerevisiae. Int. Immunopharmacol. 8, 36–42 10.1016/j.intimp.2007.10.005 18068098

[36] Murdoch C., Muthana M., Coffelt S.B. and Lewis C.E. (2008) The role of myeloid cells in the promotion of tumour angiogenesis. Nat. Rev. Cancer 8, 618–631 10.1038/nrc2444 18633355

[37] Huang L.E., Gu J., Schau M. and Bunn H.F. (1998) Regulation of hypoxia-inducible factor 1alpha is mediated by an O2-dependent degradation domain via the ubiquitin-proteasome pathway. Proc. Natl. Acad. Sci. U.S.A. 95, 7987–7992 10.1073/pnas.95.14.7987 9653127 PMC20916

[38] Epstein A.C., Gleadle J.M., McNeill L.A., Hewitson K.S., O'Rourke J., Mole D.R. et al. (2001) C. elegans EGL-9 and mammalian homologs define a family of dioxygenases that regulate HIF by prolyl hydroxylation. Cell 107, 43–54 10.1016/S0092-8674(01)00507-4 11595184

[39] Kim B.Y., Kim H., Cho E.J. and Youn H.D. (2008) Nur77 upregulates HIF-alpha by inhibiting pVHL-mediated degradation. Exp. Mol. Med. 40, 71–83 10.3858/emm.2008.40.1.71 18305400 PMC2679322

[40] Chen J., Lopez-Moyado I.F., Seo H., Lio C.J., Hempleman L.J., Sekiya T. et al. (2019) NR4A transcription factors limit CAR T cell function in solid tumours. Nature 567, 530–534 10.1038/s41586-019-0985-x 30814732 PMC6546093

[41] Yoo Y.G., Yeo M.G., Kim D.K., Park H. and Lee M.O. (2004) Novel function of orphan nuclear receptor Nur77 in stabilizing hypoxia-inducible factor-1alpha. J. Biol. Chem. 279, 53365–53373 10.1074/jbc.M408554200 15385570

[42] Kim B.Y., Kim H., Cho E.J. and Youn H.D. (2008) Nur77 upregulates HIF-alpha by inhibiting pVHL-mediated degradation. Exp. Mol. Med. 40, 71–83 10.3858/emm.2008.40.1.71 18305400 PMC2679322

