# Supplementary material for: β-glucan from Lentinus edodes inhibits breast cancer progression via the Nur77/HIF-1α axis
Source: Biosci Rep. 2020 Dec 14;40(12):BSR20201006. doi: 10.1042/BSR20201006 (PMC7736624; doi:10.1042/BSR20201006)
Supplement: Supplementary Figures S1-S7 and Tables S1-S3 [file BSR-2020-1006_supp.pdf]

Figure S1

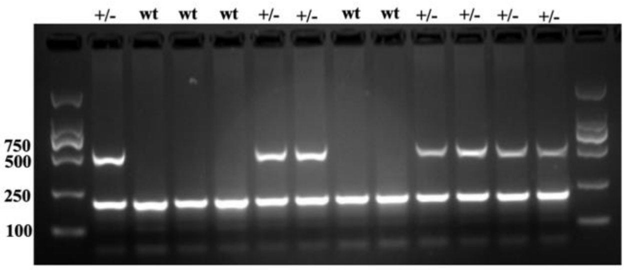

Figure S1. Genotype identification of MMTV-PyMT transgenic mice. Heterozygote (size: 556 bp and 200 bp); Wild type (size: 200 bp).

Figure S2

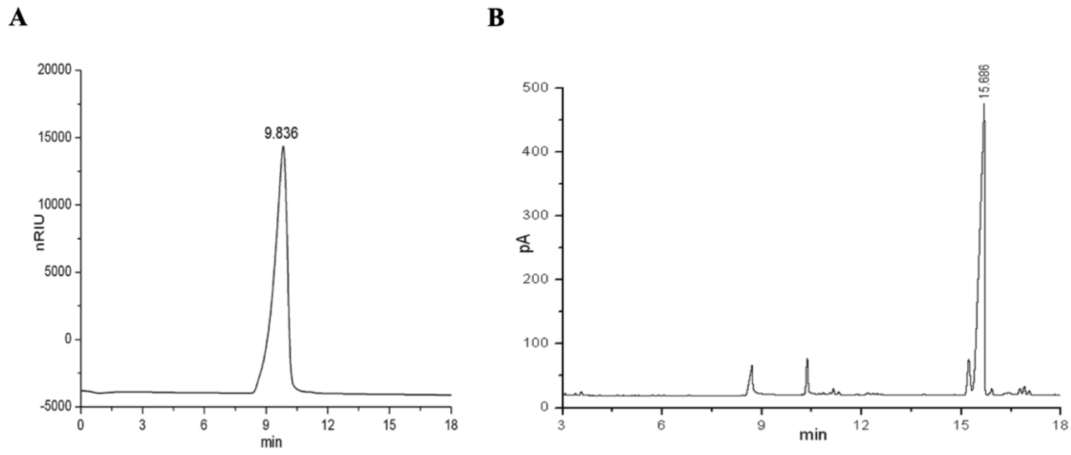

Figure S2. Identification of Lentinan (LNT). Composition (A) and monosaccharide content (B) in the mushroom polysaccharide was analyzed by HPLC/MS.

Figure S3

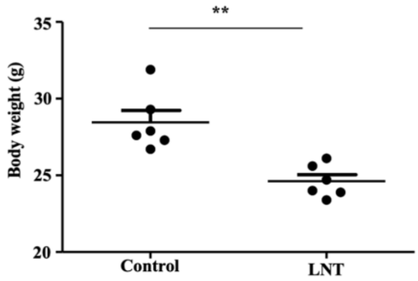

Figure S3. Body weight of each animal at the time of sacrifice.

Figure S4

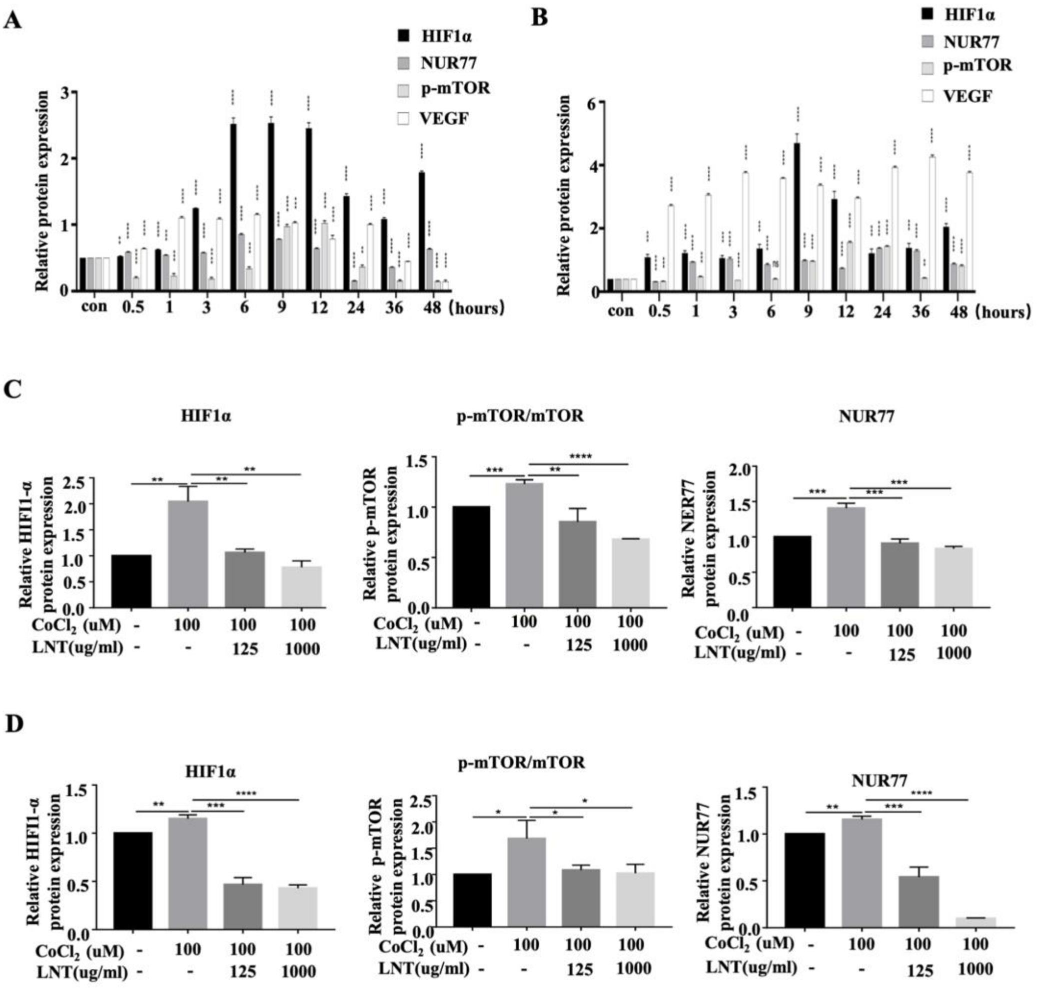

Figure S4. Quantitative analysis of protein expression in Figure 3. Quantitative analysis of HIF-1α, Nur77, VEGF and p-mTOR/mTOR ratio in panel A (A) and panel B(B) of Figure 3. Quantitative analysis of HIF-1α, Nur77 and p-mTOR/mTOR ratio in panel C (C) and panel D(D) of Figure 3.

Figure S5

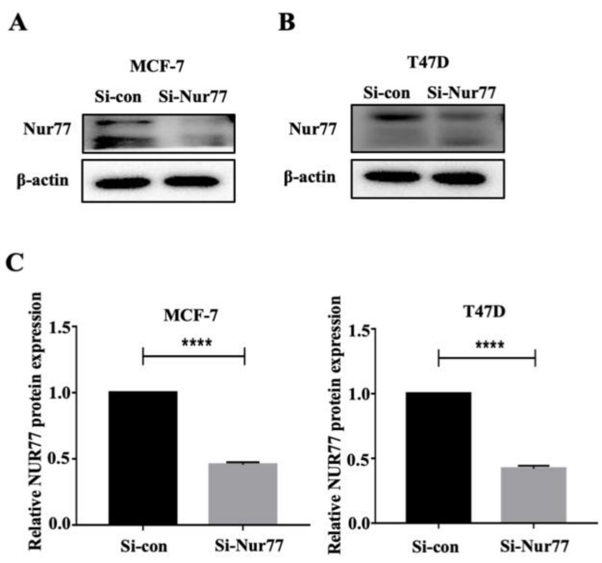

Figure S5. Identification of knockdown efficiency of Nur77. Verification of knockdown efficiency of Nur77 in (A) MCF-7 and (B) T47D cells was determined by utilizing western Blotting assay after transfecting for 48h. (C) Quantitative analysis of protein expression in panel A and B. ****, *P*<0.0001.

Figure S6

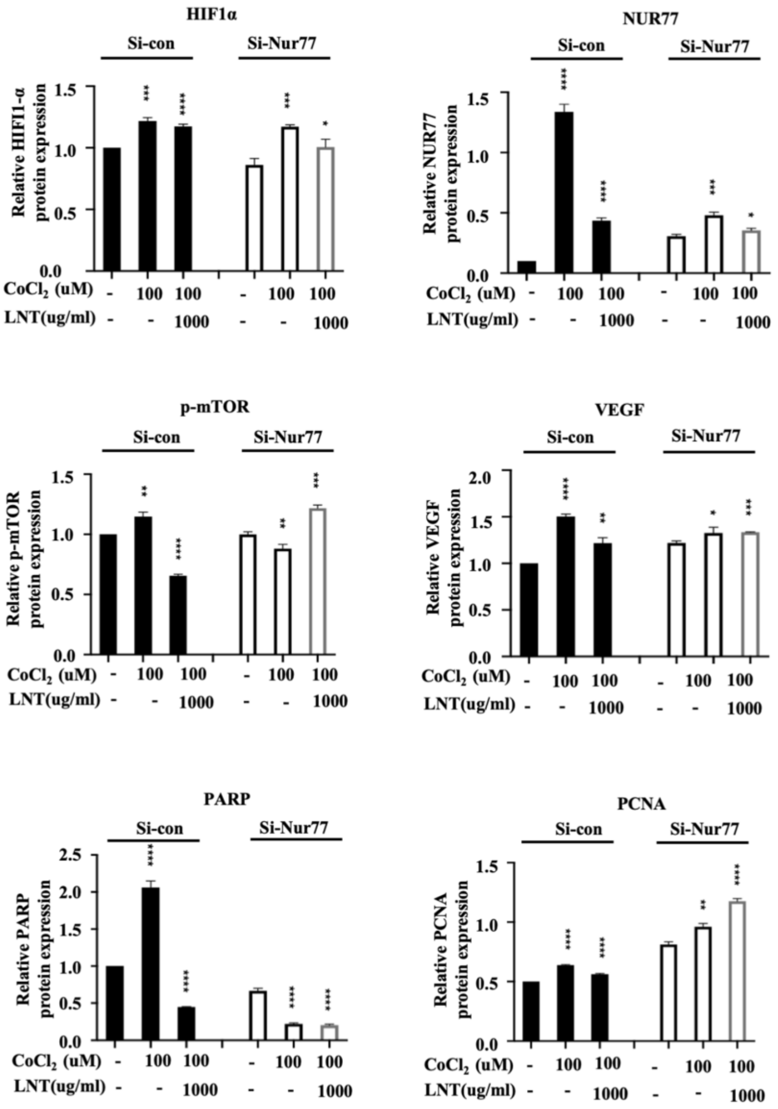

Figure S6. Quantitative analysis of protein expression in Figure 4. Quantitative analysis of HIF-1α, Nur77, VEGF, p-mTOR/mTOR ratio, PARP and PCNA in panel A of Figure 4.

Figure S7

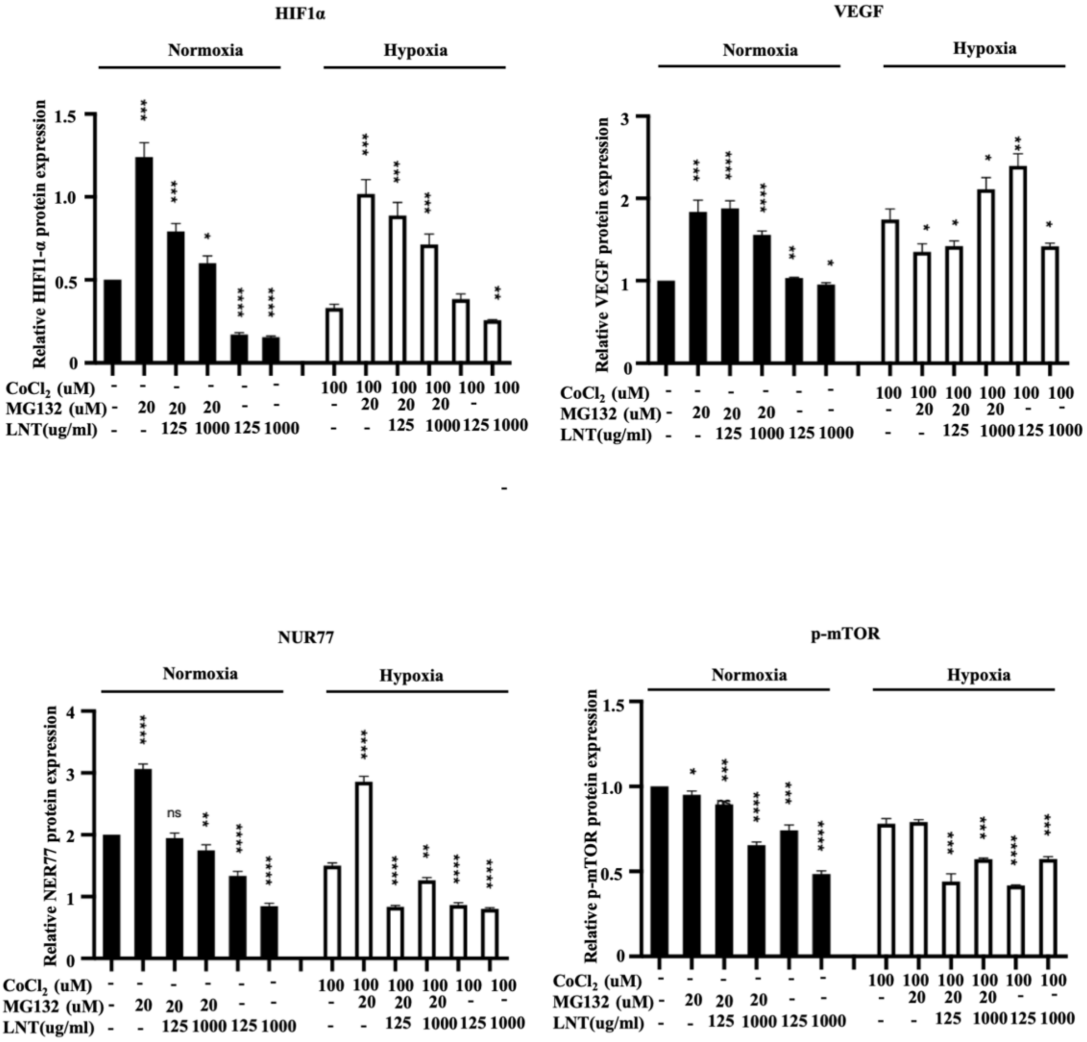

Figure S7. Quantitative analysis of protein expression in Figure 4. Quantitative analysis of HIF-1α, Nur77, VEGF and p-mTOR/mTOR ratio in panel B of Figure 4.

Table S1. Body weight at the time of sacrifice.

	Con						LNT					
Body weight (g)	29.3	31.9	27.3	27.6	27.9	26.7	24	26.1	23.4	25.6	23.9	24.7
Mean with SD	28.45±0.7753						24.62±0.4301**					

Table S2. Weight of each pair of tumors.

		Con						LNT					
Tumor weight (g)	t1	1.3	1.4	0.3	0.5	0.9	0.5	0.3	0.7	0.4	0.4	1.0	0.3
	t2	1.1	2.0	0.4	1.0	1.6	0.9	0.5	0.6	0.5	0.6	0.6	0.3
	t3	1.0	1.5	0.5	1.0	1.9	0.5	0.7	0.6	0.5	0.5	0.4	0.4
	t4	0.6	1.3	0.3	0.5	0.6	0.5	0.3	0.4	0.5	0.3	0.2	0.4
	t5	0.3	1.5	0.3	0.6	0.7	0.7	0.4	0.4	0.5	0.3	0.3	0.3
Total weight (g)		4.2	7.7	1.7	3.6	5.6	3.1	2.2	2.7	2.5	2.2	2.6	1.7
Mean with SD		4.325±0.8554						2.304±0.1497**					

t1: tumor of 1# mammary gland, t2: tumor of 2# mammary gland, t3: tumor of 3# mammary gland, t4: tumor of 4# mammary gland, t5: tumor of 5# mammary gland. 1# to 5# represented the different site of mice mammary gland (five pairs of mammary gland per mice).

Table S3. The maximum diameter of each tumor.

		Con						LNT					
Tumor diameter (cm)	t1	1.6	1.1	1.8	1	1.2	1	1.5	1.5	0.8	0.9	0.7	0.9
	t2	2	1.7	1.2	1.5	1.1	1.2	1.2	1.3	1	0.9	1	0.9
	t3	1.5	1.7	1.6	1.5	1.5	1.4	1.2	1.4	1	1	1.1	1.3
	t4	1.5	1	1	1	1	0.8	0.5	0.5	0.5	0.5	0.9	0.9
	t5	1.5	0.5	0.6	0.7	0.7	0.4	0.7	0.9	0.9	0.8	0.7	0.8
Total diameter (cm)		8.1	6	6.2	5.7	5.5	4.8	5.1	5.6	4.2	4.1	4.4	4.8
Mean with SD				6.049±0.5778						4.7±0.3985**			

t1: tumor of 1# mammary gland, t2: tumor of 2# mammary gland, t3: tumor of 3# mammary gland, t4: tumor of 4# mammary gland, t5: tumor of 5# mammary gland. 1# to 5# represented the different site of mice mammary gland (five pairs of mammary gland per mice)
